# Speciation and environmental risk of heavy metals in biochars produced by pyrolysis of chicken manure and water-washed swine manure

**DOI:** 10.1038/s41598-021-91440-8

**Published:** 2021-06-07

**Authors:** Andong Wang, Dongsheng Zou, Xinyi Zeng, Bin Chen, Xiaochen Zheng, Longcheng Li, Liqing Zhang, Zhihua Xiao, Hua Wang

**Affiliations:** 1 College of Resources and Environment, Hunan Agricultural University, Changsha, Hunan 410128 People’s Republic of China; 2 Key Laboratory for Rural Ecosystem Health in Dongting Lake Area of Hunan Province, Changsha, 410128 People’s Republic of China

**Keywords:** Environmental chemistry, Environmental impact

## Abstract

This study was conducted to investigate the speciation, bioavailability and environmental risk of heavy metals (HMs) in chicken manure (CM) and water-washed swine manure (WSM) and their biochars produced at different pyrolysis temperatures (200 to 800 °C). As the pyrolysis temperature increased, the remaining proportion, toxicity characteristic leaching procedure (TCLP), HCl and diethylenetriamine pentaacetic acid (DTPA) of HMs gradually declined. This result proved that the speciation of HMs in chicken manure biochars (CMB) and water-washed swine manure biochars (WSMB) was influenced by pyrolysis temperature. The proportions of stable fractions were enhanced with increased pyrolysis temperature and weakened the HM validity for vegetation at 800 °C. Finally, the results of the risk assessment showed that the environmental risk of HMs in CMB and WSMB decreased with increasing pyrolysis temperature. Therefore, pyrolysis at 800 °C can provide a practical approach to lessen the initial and underlying heavy metal toxicity of CMB and WSMB to the environment.

## Introduction

Over the past few decades, livestock production has experienced an industrial revolution, which has resulted in the production of mass quantities of animal manure^1^. In China, in the last few decades, animal industries have undergone a drastic conversion from traditional domestic production mainly for self-consumption or local-market sales to intensive industrial production^2^. As a result, nearly 3.2 × 10^9^ tons of livestock and poultry manure are produced annually from animal husbandry in China^3^. Livestock and poultry manure has many useful nutrients^4^, can further promote crop production^5^ and is an organic substitute for chemical fertilizers^6^. Feed, on the other hand, contains toxic HMs, which are also found in crops in farmlands where manure has been applied^7^. Therefore, suitable management and settlement of livestock manure have become of increasing concern.

Pyrolysis has become a general treatment method to deal with manure^8,9^. Pyrolysis operations can decrease the risk of environmental contamination^10,11^ and the process decreases the risk of ash cohesion^12^ and harmful discharges such as NO_X_, SO_2_, and particulates^13,14^. At the same time, pyrolysis operations allow the conversion of livestock manure into biochars^15^. Previous research has suggested that pyrolysis can probably decrease the environmental risks of using biochar in soils^16^. Meng et al. researched the physical and chemical properties of biochar produced by fresh and aerobically composted pig manure at pyrolysis temperatures of 400 °C and 700 °C and found that the ash content, surface area, pH, EC, mineral nutrients, and increased with increased temperature in the biochar^17^. Chen et al.researched the amounts of HMs, effective HMs (extracted by DTPA (diethylenetriamine pentaacetic acid)), radicals and decomposed biochar in corn stover and pig excrement and in converted biochar at 300 °C and 500 °C and found that the inorganic matter in biochar significantly immobilises the available heavy metals compared to the heavy metals in the feedstock^18^. Therefore, application of biochar from the pyrolysis of livestock manure to the soil is more beneficial than direct application of raw materials to improve the soil and reduce environmental risks. At the same time, biochar can be used as profitable products to make the process more economical^19^. The previous research shown that the utilization of biochar in the field of solid fuel, adsorbent, catalysis, etc. will significantly improve the process’s economic performance such as application of biochar as solid fuel with selling price of $50 per MT contributes to about 2.8% of the total revenue generated from the process and conversion of biochar into active carbon can significantly improve its selling price as $1188 per MT, thereby increasing its contribution to 51.11% to total revenue^20,21^.

Manure cleaning by rinsing has been a widespread method of manure disposal for dairy farms around the world^22^. In a water-washed swine manure system, large amounts of water flush the manure digested by animals, which enters a ditch along a sloping lane that then deposits the washed manure in a ballast tank or lagoon. The flushed liquid waste can also be sent to a pumping pit and pumped into a warehouse or other processing facility. Previous research has explained that flushing provides drier floors, cleaner facilities for the animals and alleviates ammonia emissions from the barnyard^23^. In addition, the flushed-manure treatment system has been widely used in large dairy farms due to the reduction of manual and mechanical failures^24^. Nevertheless, the discharge of HMs in the pyrolysis treatment of manure that has been treated by rinsing has received little attention or research.

To obtain a more extensive understanding of the contamination standards and potential ecological risks of HMs in livestock and poultry excrement and their converted biochars, the heavy metal pollution hazards associated with these materials must be systematically and quantitatively evaluated. Consequently, the main purpose of the present study is to (1) assess the bioavailability and ecological toxicity of HMs in chicken manure biochar (CMB) and water-washed swine manure biochar (WSMB) using a sequential extraction process (BCR), diethylenetriamine pentaacetic acid (DTPA), HCl leaching, and a toxicity characteristic leaching procedure (TCLP) and (2) assess potential ecological risks.

## Result and discussion

### Total heavy metal concentrations in chicken manure, water-washed swine manure, chicken manure biochar and water-washed swine manure biochar

The total HM concentrations in chicken manure (CM), water-washed swine manure (WSM) and their biochars were pyrolysis at temperatures from 200 to 800 °C, as is shown in Table 1.

**Table 1:** Total concentration of heavy metals in CM, WSM, CMB and WSMB (mg kg^−1^).

T (°C)	Zn		Cu		Ni			
	CM	WSM	CM	WSM	CM		WSM	
Material	61.47 ± 0.01	794.57 ± 1.01	24.77 ± 0.92	319.89 ± 2.25	3.87 ± 0.02		10.93 ± 0.06	
200	81.40 ± 0.02	889.34 ± 1.02	29.85 ± 0.05	359.66 ± 2.02	4.34 ± 0.03		12.88 ± 0.02	
350	103.64 ± 0.01	1589.61 ± 0.91	40.85 ± 0.01	780.35 ± 1.98	6.52 ± 0.01		25.35 ± 0.03	
500	130.85 ± 0.03	1514.73 ± 2.35	44.55 ± 0.03	937.84 ± 3.56	6.91 ± 0.00		32.02 ± 0.02	
650	130.99 ± 0.01	1752.14 ± 2.21	50.70 ± 0.02	988.79 ± 1.27	7.68 ± 0.02		33.36 ± 0.01	
800	159.58 ± 0.01	1684.02 ± 2.01	55.05 ± 0.56	989.23 ± 0.56	8.79 ± 0.01		34.23 ± 0.01	
Threshold value ^a^	500		85		-^b^			

T (°C)	Mn		Cr		As		Cd	
	CM	WSM	CM	WSM	CM	WSM	CM	WSM
Material	184.40 ± 2.03	326.29 ± 1.20	7.72 ± 0.04	4.73 ± 0.01	1.20 ± 0.02	3.15 ± 0.02	0.71 ± 0.03	0.29 ± 0.00
200	189.65 ± 1.27	359.38 ± 1.08	9.96 ± 0.03	5.49 ± 0.01	1.37 ± 0.03	3.30 ± 0.02	0.71 ± 0.01	0.27 ± 0.01
350	272.32 ± 1.28	783.99 ± 2.11	15.44 ± 0.01	9.77 ± 0.01	1.97 ± 0.01	7.11 ± 0.00	1.18 ± 0.02	0.59 ± 0.01
500	260.89 ± 2.34	938.85 ± 0.02	13.29 ± 0.01	12.47 ± 0.02	2.01 ± 0.01	8.51 ± 0.02	1.12 ± 0.02	0.77 ± 0.02
650	274.88 ± 2.56	935.23 ± 0.03	15.65 ± 0.01	11.55 ± 0.02	2.02 ± 0.02	8.89 ± 0.01	1.19 ± 0.00	0.75 ± 0.00
800	342.81 ± 1.27	1001.23 ± 0.02	17.89 ± 0.02	12.93 ± 0.01	2.10 ± 0.01	9.42 ± 0.01	1.30 ± 0.01	0.79 ± 0.01
Threshold value ^a^	-^b^		150		30		3	

Note: T: temperature, Zn: zinc, Cu: copper, Ni: nickel, Mn: manganese, Cr: chromium, As: arsenic, Cd: cadmium, CM: chicken manure, WSM: water-washed swine manure, CMB: chicken manure biochar, WSMB: water–washed swine manure biochar.

^a^From (G/BT 25,246–2010, pH < 6.5 and NY 525–2012).

^b^Not enlisted.

In general, the concentrations of Cd, Cr and As in CM and WSM were below the threshold values (technology code for land application rates of livestock and poultry manure, GBT 25246-2010, pH < 6.5 and organic fertilizer, NY 525-2012), but the Zn and Cu concentrations in WSM exceeded the threshold value. The concentrations of HMs in CM, WSM and biochars showed increasing trends as Mn > Zn > Cu > Cr > Ni > As > Cd and Zn > Mn > Cu > Ni > Cr > As > Cd, respectively.

The total concentrations of Zn, Cu and Mn in CM were 61.47, 24.77 and 184.40 mg kg^−1^, respectively. The total concentrations of Zn, Cu and Mn in WSM were significantly higher than those of the four other HMs. The total concentrations of Zn, Cu and Mn in WSM were determined to be 794.56, 319.89 and 326.29 mg kg^−1^, respectively. Previous research determined that zinc, copper, and manganese are the main trace elements in livestock and poultry manure^25^. The main trace elements contained in feed additives have been widely used in livestock and poultry feed and provide good feed conditions for the healthy growth of livestock and poultry but result in higher zinc, copper and manganese levels in CM and WSM^26^.

Table 1 shows that the HM concentrations in CMB and WSMB were all greater than those in CM and WSM. After pyrolysis, decomposition of the organic substances in manures induces the resolving of HMs linked to the organic matter and HM deposition in biochars^26^. The deposited HMs in CMB and WSMB may cause HM enrichment in CMB and WSMB. Furthermore, the weights of CM and WSM were reduced because of the disintegration of organic substances after pyrolysis^27^. The quantity decrease of organic substances was greater than that of HMs during pyrolysis and induced an increase in the HMs contents of the biochars^28^. HMs are present in manure in many forms, such as sodium hydroxide, inorganic salts, sulphides and oxides^12^. The inorganic salts and hydroxides are converted into thermally stable substances with increasing pyrolysis temperature^29^. Therefore, most HMs remain in the biochar.

**Figure 1:**
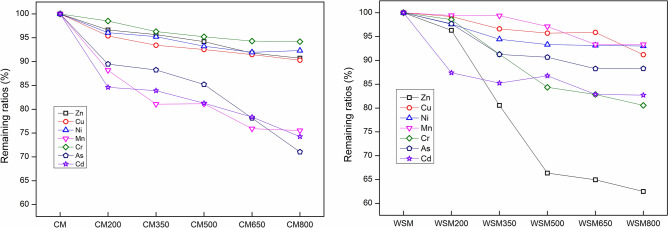
Remaining ratio: rates of heavy metal quantities in CM, WSM, CMB and WSMB. Note: Zn: zinc, Cu: copper, Ni: nickel, Mn: manganese, Cr: chromium, As: arsenic, Cd: cadmium, CM: chicken manure, WSM: water-washed swine manure, CM200-CM800: chicken manure biochar converted by pyrolysis temperatures from 200 to 800 °C, WSM200-WSM800: water-washed swine manure biochar converted by pyrolysis temperatures from 200 to 800 °C.

The R of HMs in biochars is shown in Fig. 1. For Ni, R decreased from 96.06% to 92.31% in CMB in the range from 200 to 800 °C and from 97.60% to 93.00% in WSMB in the range from 200 to 800 °C. A similar result was seen for Cu. For Cd, a conspicuous decrease was seen in CMB from 84.61% (200 °C) to 74.25% (800 °C). For Zn, a similar conspicuous decrease was seen in WSMB from 96.31% (200 °C) to 62.49% (800 °C). Similar change trends were seen for Cr and Mn. The R of HMs decreased with increasing pyrolysis temperatures was owing to volatilization tendencies of heavy metals different fractions and were possibly influenced by the reactions with an ash matrix^30^. Previous research has found that HMs, for example, Cd and Zn, can slightly constitute highly volatile compounds (e.g., ZnCl_2_ and CdCl_2_)^31,32^ and thus the R of Cd and Zn in biochar sharply decreased from 200 to 800 °C.

Additionally, the results suggested that the Rs of HMs in CMB were higher than the Rs of HMs in WSMB. The properties of CM and WSM are shown in Table 2. Previous research has found that HMs are enriched in ash with increasing pyrolysis temperature^32^ and partion of HMs was influenced by the diffusion kinetics inside the ash and by the reactions with an ash matrix^33^. In this research, most of HMs may not to diffuse out of the ash particle and trapped or reacted with the mineral constituents of the ash and then retained^34^. Therefore, due to the ash concentrations in CM were higher than the ash concentrations in WSM, which cause the Rs of HMs in WSMB to be less than the Rs of HMs in CMB. In summary, biochars with a high ash content that are produced biochars and used in the environment should attract the greatest attention.

**Table 2:** Physicochemical characteristics and elemental analyses of the manures.

Materials	Proximate analysis (wt.%)								
	Moisture	Ash	Volatile matter	Fixed carbon	N	C	H	S	O
Chicken manure	7.18	31.18	54.79	6.83	2.44	29.82	4.50	0.15	63.07
Water-washed swine manure	5.38	9.02	71.17	14.41	1.52	41.91	6.06	0.18	50.30

Note: wt: wight, N: nitrogen, C: carbon, H: hydrogen, S: sulfur, O: oxygen.

### Chemical speciation of heavy metals in chicken manure, water-washed swine manure, chicken manure biochar and water-washed swine manure biochar

The effectiveness and toxicity of HMs in the environment are based on the chemical morphology of HMs, which can be evaluated by BCR sequential extraction. The BCR results are shown in Table 3, and the heavy metals in the CM, WSM and their biochars can be grouped into four fractions: acid extractable (F1), reducible (F2), oxidizable (F3), and residual (F4)^35^. The effectiveness and redeployment of the metal fractions are in the increasing order of F1 > F2 > F3 > F4^36^.The HMs present in the F1 and F2 portions are more easily absorbed by plants and exist in surface water bodies, so they are considered to have direct toxicity and effectiveness. The F3 fraction undergoes degradation and leaching under strong acidity and strong oxidation conditions and is possibly the toxic category that presents less risk to the environment^28^. The F4 portion is regarded as non-bioavailable and nontoxic because the residual solids, which mainly contain primary and secondary solids, contain metals in their crystal structures^28,37^. Table 3 shows the BCRs of CM, WSM and the HMs in biochar produced by manure pyrolysis. The study found that pyrolysis can convert unstable components into stable components to address HMs.

**Table 3:** The speciation of heavy metals (mg kg^−1^).

HM	Fraction	Category	T (℃)					
			Materials	200	350	500	650	800
Zn	F1	CM	7.78 ± 0.01	4.81 ± 0.02	2.10 ± 0.01	0.85 ± 0.02	1.70 ± 0.01	0.55 ± 0.01
		WSM	207.08 ± 0.02	230.38 ± 0.03	415.07 ± 0.01	363.81 ± 0.01	314.88 ± 0.01	167.13 ± 0.12
	F2	CM	9.39 ± 0.02	6.32 ± 0.01	4.95 ± 0.01	4.61 ± 0.01	4.23 ± 0.13	0.67 ± 0.01
		WSM	170.75 ± 0.01	165.65 ± 0.01	155.04 ± 0.02	139.46 ± 0.03	132.48 ± 0.11	121.65 ± 0.01
	F3	CM	13.79 ± 0.03	27.97 ± 0.02	51.68 ± 0.03	56.39 ± 0.12	53.63 ± 0.23	54.28 ± 0.02
		WSM	211.30 ± 0.02	268.19 ± 0.02	468.79 ± 0.03	545.80 ± 0.12	450.86 ± 0.23	207.80 ± 0.02
	F4	CM	34.64 ± 0.1	35.62 ± 0.03	54.77 ± 0.02	58.27 ± 0.01	69.89 ± 0.12	91.00 ± 0.01
		WSM	219.60 ± 0.01	230.79 ± 0.01	585.16 ± 0.02	594.46 ± 0.01	783.37 ± 0.01	1217.48 ± 0.01
Cu	F1	CM	0.80 ± 0.03	1.51 ± 0.12	1.71 ± 0.01	2.00 ± 0.12	2.03 ± 0.01	2.15 ± 0.02
		WSM	25.68 ± 0.02	20.60 ± 0.23	16.39 ± 0.02	4.50 ± 0.03	8.35 ± 0.02	2.11 ± 0.01
	F2	CM	7.52 ± 0.12	6.72 ± 0.34	3.05 ± 0.23	2.69 ± 0.02	2.63 ± 0.00	1.87 ± 0.01
		WSM	7.72 ± 0.23	6.52 ± 0.02	5.52 ± 0.16	4.63 ± 0.01	3.05 ± 0.01	1.69 ± 0.03
	F3	CM	8.46 ± 0.01	7.32 ± 0.03	14.78 ± 0.02	9.88 ± 0.00	3.34 ± 0.02	2.14 ± 0.01
		WSM	154.95 ± 0.01	167.36 ± 0.02	404.65 ± 1.20	494.79 ± 0.01	468.06 ± 1.25	319.34 ± 0.01
	F4	CM	8.87 ± 0.01	12.94 ± 0.12	23.29 ± 2.10	31.03 ± 0.01	41.83 ± 0.38	50.17 ± 0.00
		WSM	156.70 ± 0.01	198.86 ± 0.13	404.38 ± 0.12	507.67 ± 3.14	579.56 ± 1.45	743.90 ± 0.02
Ni	F1	CM	0.33 ± 0.01	0.12 ± 0.00	0.25 ± 0.01	0.31 ± 0.01	0.19 ± 0.01	0.33 ± 0.13
		WSM	1.48 ± 0.02	0.76 ± 0.01	0.59 ± 0.02	0.60 ± 0.02	0.60 ± 0.23	1.29 ± 0.12
	F2	CM	0.64 ± 0.02	0.61 ± 0.01	0.33 ± 0.00	0.32 ± 0.00	0.31 ± 0.02	0.31 ± 0.01
		WSM	1.98 ± 0.04	1.83 ± 0.02	1.79 ± 0.01	1.61 ± 0.01	1.55 ± 0.01	1.08 ± 0.05
	F3	CM	1.07 ± 0.01	1.45 ± 0.01	1.86 ± 0.02	2.08 ± 0.03	1.39 ± 0.12	0.93 ± 0.05
		WSM	2.71 ± 0.01	2.02 ± 0.02	8.21 ± 0.01	11.61 ± 0.14	12.78 ± 1.23	7.51 ± 0.01
	F4	CM	1.92 ± 0.02	2.34 ± 0.01	4.38 ± 0.01	4.46 ± 0.13	5.92 ± 0.32	7.41 ± 1.45
		WSM	4.35 ± 0.10	7.19 ± 0.12	14.18 ± 0.03	16.25 ± 1.10	16.45 ± 0.27	23.30 ± 2.31
Mn	F1	CM	28.29 ± 2.01	23.09 ± 1.56	22.14 ± 0.23	21.46 ± 0.01	20.85 ± 1.11	20.39 ± 5.21
		WSM	65.24 ± 3.12	64.80 ± 4.32	128.20 ± 1.11	120.37 ± 0.01	122.36 ± 1.54	132.10 ± 1.22
	F2	CM	26.26 ± 2.14	25.57 ± 0.06	24.67 ± 0.01	22.53 ± 2.31	20.00 ± 0.21	8.72 ± 0.01
		WSM	78.48 ± 0.23	77.45 ± 0.23	75.05 ± 0.03	74.36 ± 2.13	75.45 ± 2.31	72.64 ± 0.23
	F3	CM	47.01 ± 0.35	49.53 ± 0.47	79.53 ± 0.02	96.18 ± 0.01	95.59 ± 7.65	94.16 ± 0.12
		WSM	80.00 ± 0.01	81.40 ± 0.01	197.40 ± 0.03	297.53 ± 0.23	288.38 ± 8.23	287.86 ± 0.56
	F4	CM	72.55 ± 0.01	82.76 ± 0.01	128.89 ± 0.01	134.48 ± 0.01	147.63 ± 0.01	200.40 ± 1.58
		WSM	89.99 ± 0.01	134.78 ± 3.21	376.51 ± 0.21	440.90 ± 0.01	451.30 ± 0.01	500.44 ± 7.32
Cr	F1	CM	0.96 ± 0.01	0.53 ± 0.01	0.15 ± 0.02	0.08 ± 0.01	0.07 ± 0.01	0.22 ± 0.02
		WSM	0.07 ± 0.01	0.09 ± 0.00	0.16 ± 0.03	0.07 ± 0.02	0.11 ± 0.01	0.11 ± 0.01
	F2	CM	0.31 ± 0.02	0.29 ± 0.06	0.14 ± 0.01	0.01 ± 0.00	0.01 ± 0.00	0.01 ± 0.00
		WSM	0.11 ± 0.01	0.11 ± 0.01	0.11 ± 0.00	0.11 ± 0.00	0.10 ± 0.02	0.10 ± 0.00
	F3	CM	1.56 ± 0.02	1.15 ± 0.05	2.37 ± 0.12	3.34 ± 1.21	1.39 ± 0.23	0.91 ± 0.16
		WSM	2.11 ± 0.04	2.65 ± 0.03	4.37 ± 0.13	5.02 ± 1.23	4.83 ± 1.10	2.78 ± 0.23
	F4	CM	4.50 ± 0.10	6.54 ± 0.12	10.12 ± 1.20	10.14 ± 2.35	13.40 ± 3.21	15.87 ± 0.01
		WSM	2.77 ± 0.23	2.88 ± 0.45	6.87 ± 1.21	7.88 ± 0.03	8.38 ± 2.89	10.83 ± 0.01
As	F1	CM	0.27 ± 0.01	0.17 ± 0.02	0.35 ± 0.01	0.17 ± 0.03	0.21 ± 0.03	0.14 ± 0.01
		WSM	0.42 ± 0.02	0.47 ± 0.03	0.39 ± 0.01	0.29 ± 0.03	0.26 ± 0.02	0.27 ± 0.02
	F2	CM	0.15 ± 0.01	0.14 ± 0.01	0.13 ± 0.01	0.13 ± 0.02	0.11 ± 0.01	0.08 ± 0.01
		WSM	0.58 ± 0.10	0.56 ± 0.01	0.53 ± 0.03	0.51 ± 0.12	0.50 ± 0.12	0.41 ± 0.10
	F3	CM	0.17 ± 0.01	0.16 ± 0.01	0.55 ± 0.02	0.45 ± 0.12	0.59 ± 0.01	0.57 ± 0.11
		WSM	1.05 ± 0.02	1.02 ± 0.23	3.35 ± 0.01	3.53 ± 0.01	3.51 ± 0.01	3.76 ± 0.01
	F4	CM	0.57 ± 0.12	0.75 ± 0.03	0.82 ± 0.01	1.16 ± 0.11	1.04 ± 0.02	1.23 ± 0.02
		WSM	1.06 ± 0.12	1.44 ± 0.02	2.81 ± 0.02	4.33 ± 0.10	4.53 ± 0.01	4.88 ± 1.20
Cd	F1	CM	_^a^	0.01 ± 0.00	_^a^	_^a^	0.01 ± 0.00	0.01 ± 0.00
		WSM	0.05 ± 0.01	0.04 ± 0.00	0.11 ± 0.01	0.04 ± 0.01	0.05 ± 0.00	0.02 ± 0.00
	F2	CM	0.10 ± 0.01	0.08 ± 0.02	0.08 ± 0.01	0.07 ± 0.01	0.07 ± 0.01	0.06 ± 0.01
		WSM	0.03 ± 0.00	0.03 ± 0.01	0.03 ± 0.00	0.02 ± 0.00	0.02 ± 0.00	0.01 ± 0.00
	F3	CM	0.11 ± 0.01	0.11 ± 0.02	0.16 ± 0.01	0.16 ± 0.02	0.15 ± 0.04	0.13 ± 0.05
		WSM	0.09 ± 0.00	0.08 ± 0.03	0.22 ± 0.02	0.33 ± 0.01	0.30 ± 0.05	0.13 ± 0.03
	F4	CM	0.51 ± 0.01	0.52 ± 0.01	0.85 ± 0.10	0.90 ± 0.01	0.99 ± 0.14	1.12 ± 0.02
		WSM	0.10 ± 0.02	0.13 ± 0.01	0.24 ± 0.12	0.36 ± 0.04	0.38 ± 0.10	0.63 ± 0.01

Note: HM: heavy metal, T: temperature, Zn: zinc, Cu: copper, Ni: nickel, Mn: manganese, Cr: chromium, As: arsenic, Cd: cadmium, CM: chicken manure, WSM: water-washed swine manure, CMB: chicken manure biochar, WSMB: water–washed swine manure biochar, F1: acid extractable, F2: reducible, F3: oxidizable, F4: residual.

a Below the detection limits.

**Figure 2:**
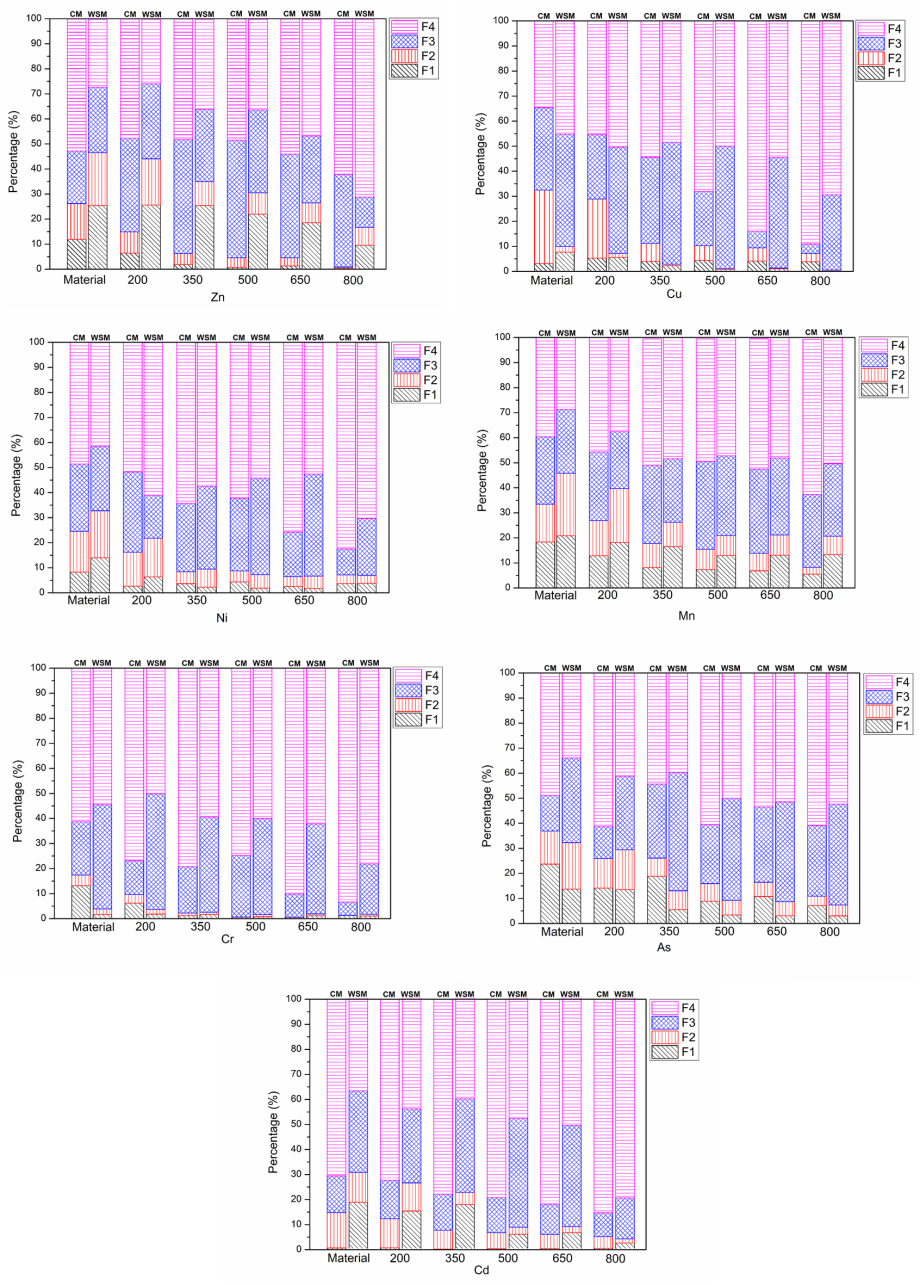
Percentages of fractions of heavy metals in CM, WSM, CMB and WSMB. Note: Zn: zinc, Cu: copper, Ni: nickel, Mn: manganese, Cr: chromium, As: arsenic, Cd: cadmium, CM: chicken manure, WSM: water-washed swine manure, CMB: chicken manure biochar, WSMB: water–washed swine manure biochar, F1: acid extractable, F2: reducible, F3: oxidizable, F4: residual.

Figure 2 shows the percentage of Zn, Cu, Ni, Mn, Cr, As and Cd in CM, WSM, CMB and WSMB. HMs except for Cd, Cr and Ni in CM and CMB. As well as, except Cu and Cr in WSM and WSMB. More than thirty percent of the Zn, Cu, Mn and As in CM and CMB were in the bioavailable portion (e.g., F1 + F2). Additionally, more than thirty percent of the Cd, Zn, Ni, Mn and As in WSM and WSMB were in the bioavailable portion (F1 + F2). These results demonstrate the higher environmental risk if the samples are placed into the ground. A clear decrease was present in the direct and bioavailable portions after the manure was converted into biochar after pyrolysis. With increasing pyrolysis temperatures from 200 to 800 °C, the percentage of the bioavailable portion (e.g., F1 + F2) in the biochars declined and a stable increase appeared in the percentage of the residual portion (F4) in biochars. Other previous studies have found similar results^3,38^. The percent of unstable fractions (F1 + F2) declined and an increasing trend appeared in the percent of the stable fractions (F3 + F4) with increased pyrolysis temperature. For example, for Zn, the percent of unstable fractions (F1 + F2) decreased from 26.17 (CM) to 0.84% (CMB) and from 46.71 (WSM) to 16.84% (WSMB) with increased pyrolysis temperature. The percent of the stable fractions (F3 + F4) increased from 73.82 (CM) to 99.15% (CMB) and from 53.28 (WSM) to 83.15% (WSMB) with increased pyrolysis temperature. For Cu, the percent of unstable fractions (F1 + F2) decreased from 32.81 (CM) to 7.13% (CMB) and from 9.67 (WSM) to 0.35% (WSMB) with increased pyrolysis temperature. The percent of the stable fraction (F3 + F4) increased from 68.36 (CM) to 92.86% (CMB) and from 90.32 (WSM) to 99.64% (WSMB) with increased pyrolysis temperature. For Ni, the percent of unstable portions (F1 + F2) decreased from 24.49 (CM) to 7.14% (CMB) and from 32.90 (WSM) to 7.15% (WSMB) with increased pyrolysis temperature. The percent of the stable fractions (F3 + F4) increased from 75.50 (CM) to 92.85% (CMB) and from 67.09 (WSM) to 92.84% (WSMB) with increased pyrolysis temperature. For Mn, the percent of unstable portions (F1 + F2) declined from 31.32 (CM) to 8.99% (CMB) and from 45.81 (WSM) to 20.61% (WSMB) with increased pyrolysis temperature. The percent of the stable portions (F3 + F4) increased from 68.67 (CM) to 91.00% (CMB) and from 54.18 (WSM) to 79.38% (WSMB) with increased pyrolysis temperature. For Cr, the percent of unstable portions (F1 + F2) declined from 17.38 (CM) to 1.31% (CMB) and from 3.61 (WSM) to 1.51% (WSMB) with increased pyrolysis temperature. The percent of the stable fractions (F3 + F4) increased from 82.61 (CM) to 98.68% (CMB) and from 96.38 (WSM) to 98.48% (WSMB) with increased pyrolysis temperature. For As, the percent of unstable portions (F1 + F2) declined from 36.84 (CM) to 10.89% (CMB) and from 32.20 (WSM) to 7.30% (WSMB) with increased pyrolysis temperature. The percent of the stable fractions (F3 + F4) increased from 63.15 (CM) to 89.10% (CMB) and from 67.79 (WSM) to 92.69% (WSMB) with increased pyrolysis temperature. For Cd, the percent of unstable portions (F1 + F2) declined from 14.83 (CM) to 5.21% (CMB) and from 30.38 (WSM) to 3.83% (WSMB) with increased pyrolysis temperature. The percent of the stable fractions (F3 + F4) increased from 85.16 (CM) to 94.78% (CMB) and 69.61 (WSM) to 96.16% (WSMB) with increased pyrolysis temperature. With increased pyrolysis temperatures, HMs can preferentially combine with organic matter in the residue to form stable fractions^39^. In comparison with the raw materials, the heavy metal concentrations in the residues increased due to the decrease in volume during pyrolysis^40^. The volatilization rates of HMs were different at different temperatures. These variations are related to the properties of the HMs and their compounds. Therefore, the low-volatility HMs (e.g., Mn, Cr, Ni and Cu) were more likely to remain in the biochars compared with the medium-volatility Zn and Cd^41^.

With increasing pyrolysis temperatures, Zn, Ni, Mn, Cu, As and Cd are inclined to constitute the F3 portion. The F4 portion of Zn decreased from 52.79 to 47.67% in CMB when the pyrolysis temperature was 200 °C. The F4 fraction of Ni decreased from 64.32 to 62.25% in CMB when the pyrolysis temperature changed from 350 °C to 500 °C and from 60.95 to 57.24% in WSMB when the pyrolysis temperature changed from 200 °C to 350 °C. The F4 fraction of Mn decreased from 50.50 to 48.96% in CMB when the pyrolysis temperature changed from 350 °C to 500 °C and from 48.44 to 47.24% in WSMB when the pyrolysis temperature was between 350 °C and 500 °C. The F4 fraction of Cu decreased from 50.55 to 48.66% in WSMB when the pyrolysis temperature changed from 200 °C to 350 °C. The F4 fraction of As decreased from 61.34 to 44.33% in CMB when the pyrolysis temperature changed from 200 °C to 350 °C and from 41.35 to 39.74% in WSMB when the pyrolysis temperature changed from 200 °C to 350 °C. The F4 fraction of Cd decreased from 44.25 to 42.28% in WSMB when the pyrolysis temperature changed from 200 °C to 350 °C. The significantly increased F3 fraction may be due to the complexation of HMs and the fixation of HMs with organic matter as pyrolysis temperatures increase^42^.

As the pyrolysis temperatures continue to increase, the percentage of Zn, Ni, Mn, Cu, As and Cd in the F4 fraction increased (Zn increased from 47.67 to 48.25% in CMB when the pyrolysis temperature rose from 200 to 350 °C. Ni increased from 62.25 to 75.74% in CMB when the pyrolysis temperature rose from 500 to 650 °C and increased from 52.44 to 70.20% in WSMB when the pyrolysis temperature rose from 650 to 800 °C. Mn increased from 48.96 to 51.96% in CMB when the pyrolysis temperature rose from 500 to 650 °C and increased from 47.24 to 48.13% in WSMB when the pyrolysis temperature rose from 500 to 650 °C. Cr increased from 74.74 to 90.54% in CMB when the pyrolysis temperature rose from 500 to 650 °C and increased from 50.35 to 56.69% in WSMB when the pyrolysis temperature rose from 200 to 350 °C. As enhanced from 44.33 to 80.49% in CMB when the pyrolysis temperature rose from 350 to 500 °C and increased from 39.74 to 50.04% in WSMB when the pyrolysis temperature rose from 350 to 500 °C. Cd increased from 40.28 to 48.09% in WSMB when the pyrolysis temperature rose from 350 to 500 °C.) Similar results have been reported in other previous research^39^. Lower pyrolysis temperatures cannot attain the boiling point of HMs^43^. Since these HMs are less volatile, it is not possible to convert most of the F3 fraction to the F4 fraction^44^. Previous research has shown that high pyrolysis temperatures correspond to adequate energy for breaking the associated bonds. Therefore, the F4 fraction of metals attained its greatest content at high temperatures^39^. The current research has shown that increased pyrolysis temperatures are conducive to creating more stable fractions.

In conclusion, pyrolysis can effectively alleviate HM concentrations in the pyrolysis residue and mitigate environmental risk.

### Toxicity characteristic leaching procedure

The results of TCLP for the HMs in CM, WSM and their biochars are shown in Table 4. The leaching potential of HMs in CMB and WSMB decreased with increased pyrolytic temperatures from 200 to 800 °C.

**Table 4:** The leachable heavy metals in CM, WSM, CMB and WSMB based on TCLP (mg kg^−1^).

T (°C)	Zn		Cu		Ni		Mn		Cr		As		Cd	
	CM	WSM	CM	WSM	CM	WSM	CM	WSM	CM	WSM	CM	WSM	CM	WSM
Material	61.16 ± 0.01	481.11 ± 1.23	24.48 ± 1.10	40.65 ± 1.78	3.29 ± 0.56	3.13 ± 0.02	163.81 ± 6.23	193.47 ± 1.23	1.31 ± 0.23	2.20 ± 0.23	1.00 ± 0.01	2.35 ± 0.02	0.23 ± 0.01	0.28 ± 0.01
200	56.96 ± 0.02	387.63 ± 2.31	24.38 ± 1.23	36.92 ± 1.35	3.24 ± 0.12	2.39 ± 0.23	163.40 ± 1.78	116.12 ± 0.02	0.74 ± 0.01	1.06 ± 0.02	0.95 ± 0.02	2.29 ± 0.01	0.22 ± 0.01	0.25 ± 0.01
350	56.60 ± 0.01	350.07 ± 1.13	22.06 ± 1.56	26.35 ± 2.34	2.98 ± 0.32	1.56 ± 0.01	161.49 ± 2.49	92.12 ± 0.02	0.27 ± 0.00	0.89 ± 0.01	0.52 ± 0.04	1.94 ± 0.02	0.20 ± 0.02	0.22 ± 0.10
500	42.44 ± 0.12	188.73 ± 1.12	21.01 ± 2.31	5.58 ± 0.23	2.91 ± 0.65	0.64 ± 0.02	148.23 ± 2.22	81.32 ± 1.21	0.22 ± 0.02	0.65 ± 0.03	0.25 ± 0.01	0.53 ± 0.03	0.19 ± 0.00	0.22 ± 0.01
650	21.55 ± 0.12	124.27 ± 1.10	19.03 ± 1.65	2.77 ± 0.56	1.93 ± 0.89	0.58 ± 0.13	132.16 ± 1.11	40.16 ± 1.11	0.18 ± 0.01	0.40 ± 0.11	0.20 ± 0.01	0.47 ± 0.01	0.11 ± 0.00	0.18 ± 0.02
800	15.92 ± 0.13	15.79 ± 0.01	8.54 ± 2.31	2.57 ± 0.91	0.07 ± 0.01	0.35 ± 0.01	126.37 ± 2.10	6.94 ± 0.23	0.11 ± 0.00	0.36 ± 0.03	0.09 ± 0.01	0.21 ± 0.01	0.08 ± 0.01	0.15 ± 0.01
Threshold value^a^	5		-^b^		5		-^b^		5		5		1	

Note: T: temperature, Zn: zinc, Cu: copper, Ni: nickel, Mn: manganese, Cr: chromium, As: arsenic, Cd: cadmium, CM: chicken manure, WSM: water-washed swine manure, CMB: chicken manure biochar, WSMB: water–washed swine manure biochar.

a From (EPA US, 1992).

b Not enlisted.

**Figure 3:**
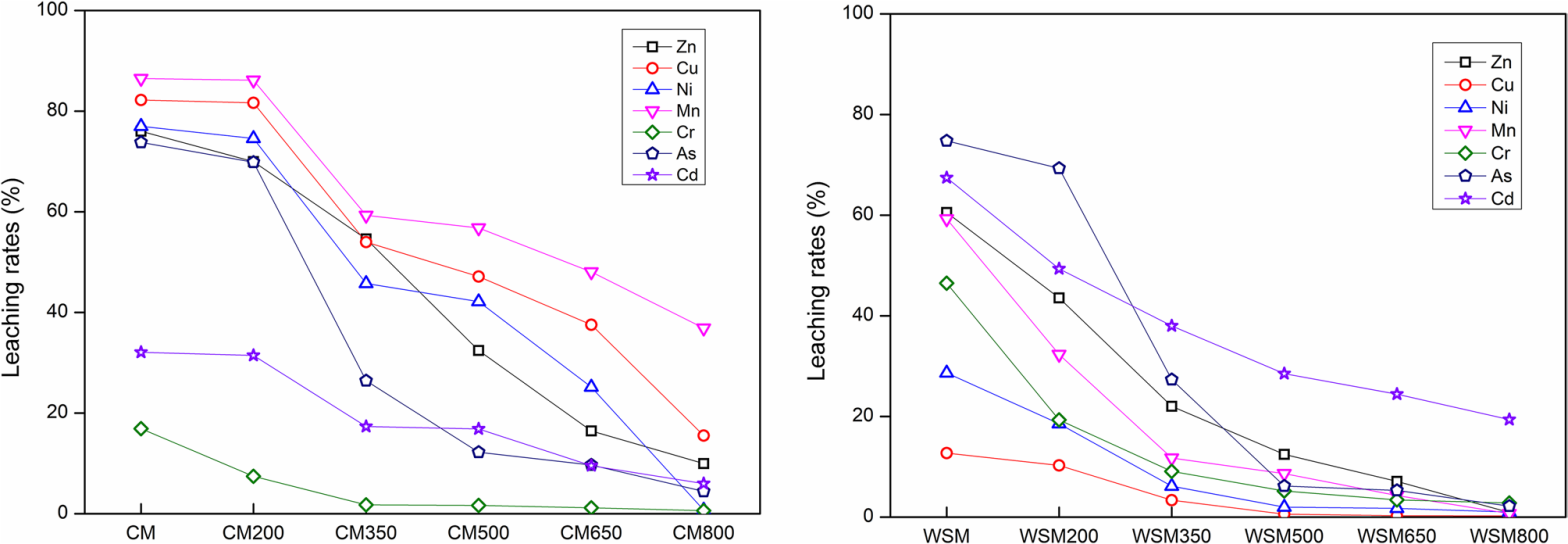
The leaching rates of heavy metals in CM, WSM, CMB and WSMB. Note: Zn: zinc, Cu: copper, Ni: nickel, Mn: manganese, Cr: chromium, As: arsenic, Cd: cadmium, CM: chicken manure, WSM: water-washed swine manure, CM200-CM800: chicken manure biochar converted by pyrolysis temperatures from 200 to 800 °C, WSM200-WSM800: water-washed swine manure biochar converted by pyrolysis temperatures from 200 to 800 °C.

The proportions of single heavy metal concentrations in the leached liquor to the entire concentration of those HMs are regarded as leachable grades^32^, and the criteria for Zn, Cu, Ni, Mn, Cr, As and Cd are shown in Fig. 3. The leaching rates of HMs in CNB and WSMB were significantly lower than the leaching rates for CM and WSM. The leaching rates of Mn, Cu, Ni, Zn and As in CMB exhibited a sharp decline. The leaching rates of As, Cd, Zn, Mn and Cr in WSMB exhibited the same trend. Previous studies have shown that each metal type in a solid matrix has a diverse affinity for particles, which results in differing release and leaching properties of Zn, Cu, Ni, Mn, Cr, As, and Cd^45,46^. In addition, the chemical and geochemical properties strongly influence metal stabilization^46,47^. Previous research has shown that the pH buffering ability in the biochar residues produced from manure was facilitated by higher pyrolysis temperatures^29^. The sharp decline in TCLP contents of HMs in CM, WSM, CMB and WSMB may rely on the reforming of the buffering ability of the biochar when temperatures increase to certain levels. Some researchers have suggested that the surface area of the biochar increased to approximately 40 m^2^ g^−1^ when the temperature was increased to 700 °C^29^. This adsorption ability of biochar may be another cause of suppressed metal leaching when the temperature increases to certain levels. Briefly, the TCLP content of CM, WSM and their biochars declined with increasing pyrolysis temperatures. These results show that the discharge of HMs greatly declined with increasing pyrolysis temperatures. Pyrolysis has a crucial impact on moderating the leaching risk of HMs in biochars produced from CM and WSM.

### Bioavailable fraction of heavy metals in manures and their biochars

The bioavailability of HMs represents those portions that are easily absorbed by plants. The DTPA-extractable part has been utilized to assess the bioavailability of HMs in excrement because of its capacity for chelating multiple metal elements^48^. To prevent the extra dissolution of CaCO_3_, the DTPA-extractable experiment was designed to extract only the soluble and exchangeable portions of contaminated metals^32^. The HCl extract was extracted by 0.1 mol l^−1^ HCl slaking to melt soluble oxides and carbonates. Next, 0.1 mol l^−1^ HCl extracted HMs that were combined with these parts and were also soluble and exchangeable.

The variations of extracted HMs concentrations express the bioavailability of HMs in biochar for pyrolysis temperatures from 200 to 800 °C. The results shown in Table 5 indicate that a similar trend is present for the HM concentrations leached by DTPA and HCl. The concentrations of plant-available HMs in CMB and WSMB decreased with increasing pyrolytic temperatures. The Zn, Cu and Mn contents could be due to soil nutrient pointers and soil environment and health pointers^49^. The Zn, Cu and Mn concentrations in CM and WSM and their biochars were comparable with soil nutrient pointers and soil environment and health pointers. Zn extracted by DTPA in CM and WSM was 33 times (CM) and 98 times (WSM) that of soil nutrient pointers and soil environmental health pointers, respectively. Cu extracted by DTPA in CM and WSM was 4 times (CM) and 40 times (WSM) that of soil nutrient pointers and soil environment and health pointers, respectively. Mn extracted by DTPA in CM and WSM was 2 times (CM) and 2 times (WSM) that of soil nutrient pointers and soil environment and health pointers, respectively. Zn, Cu and Mn leached by HCl showed the same trend. Zn extracted by HCl in CM and WSM was 20 times (CM) and 210 times (WSM) that of soil nutrient pointers and soil environment and health pointers, respectively. Cu extracted by HCl in CM and WSM was 4 times (CM) and 90 times (WSM) that of soil nutrient pointers and soil environment and health pointers, respectively. Mn extracted by HCl in CM and WSM was 33 times (CM) and 6 times (WSM) that of soil nutrient pointers and soil environment and health pointers, respectively. When pyrolysis temperatures reach 800 °C, the heavy metal contents of Zn (CMB), Cu (WSMB) and Mn (CMB and WSMB) extracted by DTPA were less than those of soil nutrient pointers and soil environment and health pointers. Similarly, when pyrolysis temperatures reached 800 °C, the heavy metal concentrations of Cu (CMB) and Mn (CMB) extracted by HCl were less than those of soil nutrient pointers and soil environment and health pointers. The alterations of bioavailable fraction of Zn, Cu and Mn in the biochars after pyrolysis may be related to the mineral components, surface areas, and surface functional groups of biochars^50–52^. The biochars acquired after pyrolysis had high concentrations of soluble P minerals^17,53^, which act as the adsorption medium of Cu^52^. The previous research also found that the mineral components and oxygen functional groups in manure biochars produced at low temperature had high adsorption capacity for Zn^50^. The mineral components and functional groups onto the biochar might reduce the release of heavy metals by chemical extraction reagent^51^. However, other HMs contents at 800 °C were greater than those of soil nutrient pointers and soil environment and health pointers. Therefore, HMs in biochars should attract wide attention. At the same time, the results showed that pyrolysis temperatures of 800 °C lower the availability of biochar HMs to plants and reduce the environmental risk of HMs.

**Table 5:** The phytoaccessible metals in CM, WSM, CMB and WSMB based on DTPA and HCl (mg kg^−1^).

T (°C)	Zn		Cu		Ni		Mn		Cr		As		Cd	
DTPA	CM	WSM	CM	WSM	CM	WSM	CM	WSM	CM	WSM	CM	WSM	CM	WSM
Material	40.18 ± 1.22	295.58 ± 1.35	8.51 ± 0.98	89.23 ± 2.34	3.08 ± 0.56	2.62 ± 0.23	74.18 ± 1.56	60.09 ± 5.20	1.40 ± 0.23	1.27 ± 0.12	0.98 ± 0.25	0.53 ± 0.01	0.21 ± 0.02	0.12 ± 0.01
200	39.29 ± 1.32	288.62 ± 2.22	6.83 ± 1.23	86.45 ± 4.32	2.38 ± 0.01	2.39 ± 0.26	56.06 ± 2.35	29.31 ± 1.20	0.98 ± 0.01	1.23 ± 0.01	0.88 ± 0.11	0.46 ± 0.02	0.23 ± 0.01	0.10 ± 0.01
350	29.77 ± 0.23	15.68 ± 1.56	5.24 ± 0.23	7.79 ± 0.23	1.98 ± 0.23	0.71 ± 0.36	47.80 ± 1.23	8.55 ± 0.89	0.63 ± 0.02	0.59 ± 0.12	0.56 ± 0.01	0.43 ± 0.00	0.20 ± 0.00	0.08 ± 0.02
500	20.04 ± 0.15	7.97 ± 0.78	4.79 ± 0.56	7.47 ± 0.45	0.95 ± 0.35	0.44 ± 0.01	39.55 ± 1.56	3.61 ± 0.23	0.36 ± 0.02	0.38 ± 0.01	0.22 ± 0.02	0.25 ± 0.01	0.14 ± 0.02	0.07 ± 0.02
650	8.56 ± 0.25	7.76 ± 0.23	3.48 ± 0.37	1.19 ± 0.12	0.92 ± 0.41	0.27 ± 0.02	37.87 ± 0.23	3.54 ± 0.12	0.35 ± 0.00	0.37 ± 0.02	0.14 ± 0.02	0.24 ± 0.1	0.02 ± 0.00	0.04 ± 0.00
800	2.18 ± 0.98	6.15 ± 0.12	2.77 ± 0.01	0.69 ± 0.32	0.50 ± 0.02	0.02 ± 0.01	25.45 ± 0.25	1.54 ± 0.14	0.32 ± 0.01	0.35 ± 0.01	0.08 ± 0.01	0.11 ± 0.01	0.01 ± 0.00	0.02 ± 0.01
HCl	CM	WSM	CM	WSM	CM	WSM	CM	WSM	CM	WSM	CM	WSM	CM	WSM
Material	61.21 ± 2.35	651.58 ± 0.12	8.40 ± 0.12	192.29 ± 0.12	2.19 ± 0.02	4.08 ± 0.01	117.47 ± 1.23	172.27 ± 2.34	2.10 ± 0.01	2.61 ± 0.01	0.95 ± 0.01	1.23 ± 0.10	0.37 ± 0.02	0.24 ± 0.01
200	58.57 ± 1.36	623.87 ± 1.89	7.26 ± 0.58	111.64 ± 0.23	1.96 ± 0.01	3.20 ± 0.12	105.92 ± 0.23	165.38 ± 3.12	1.05 ± 0.02	2.01 ± 0.05	0.95 ± 0.01	1.05 ± 0.02	0.28 ± 0.01	0.22 ± 0.01
350	42.48 ± 1.56	558.31 ± 6.23	5.91 ± 2.36	111.34 ± 0.12	0.95 ± 0.02	1.81 ± 0.03	102.41 ± 0.22	120.60 ± 0.12	0.81 ± 0.01	1.52 ± 0.02	0.50 ± 0.03	0.82 ± 0.01	0.27 ± 0.01	0.21 ± 0.02
500	39.50 ± 0.88	276.80 ± 0.01	5.94 ± 0.12	55.80 ± 0.12	0.91 ± 0.03	0.99 ± 0.05	94.70 ± 4.23	102.20 ± 0.32	0.76 ± 0.01	1.26 ± 0.01	0.19 ± 0.02	0.80 ± 0.02	0.17 ± 0.02	0.18 ± 0.02
650	34.97 ± 0.56	274.50 ± 0.01	4.16 ± 0.01	42.80 ± 0.23	0.66 ± 0.25	0.83 ± 0.06	72.59 ± 1.23	91.19 ± 0.23	0.55 ± 0.02	0.90 ± 0.03	0.20 ± 0.01	0.58 ± 0.01	0.06 ± 0.02	0.16 ± 0.02
800	9.32 ± 0.23	256.90 ± 4.56	1.68 ± 0.01	33.50 ± 0.12	0.58 ± 0.01	0.51 ± 0.01	0.87 ± 0.01	83.38 ± 1.23	0.42 ± 0.01	0.72 ± 0.02	0.16 ± 0.01	0.54 ± 0.01	0.04 ± 0.00	0.07 ± 0.00
Pyto-available heavy metals^a^	3		1.8		-^b^		30		-^b^		-^b^		-^b^	

Note: T: temperature, Zn: zinc, Cu: copper, Ni: nickel, Mn: manganese, Cr: chromium, As: arsenic, Cd: cadmium, CM: chicken manure, WSM: water-washed swine manure, CMB: chicken manure biochar, WSMB: water–washed swine manure biochar, DTPA: diethylenetriamine pentaacetic acid, HCl: hydrochloric acid.

a Yang, Z., Yu, T., Hou, Q., Xia, X., Feng, H., Huang, C., Wang, L., Lv, Y., Zhang, M. 2014. Geochemical evaluation of land quality in China and its applications. Journal of Geochemical Exploration, 139, 122–135.

b Not enlisted.

### Environmental risk assessment of heavy metals in feedstocks and their biochars

**Figure 4:**
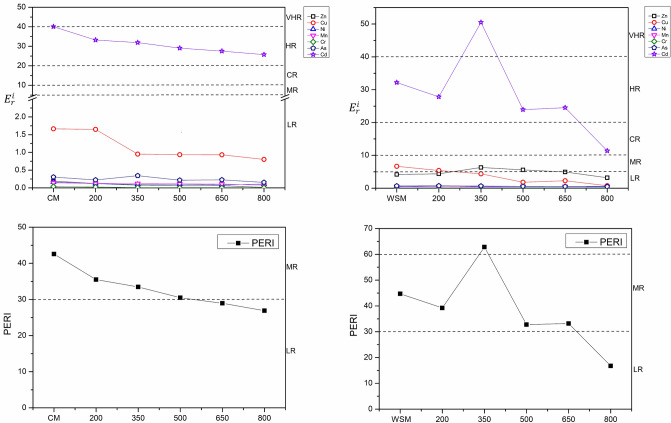
Environmental risk assessment of heavy metals in CM, WSM, CMB and WSMB. Note: environment risk index, PERI: potential ecological risks index, CM: chicken manure, WSM: water-washed swine manure.

The PERI (potential ecological risk index) value of HMs in CM, WSM, CMB and WSMB are shown in Fig. 4. The results suggest that the risk index ($E_{r}^{i}$) values for Zn, Cu, Ni, Mn, Cr and As in CM and CMB are all under 5, which indicates low risk (LR). However, the risk index ($E_{r}^{i}$) values of Cd in CM and CMB are between 40.08 and 25.74. This result shows that the risk indices ($E_{r}^{i}$) for the local environment changed from very high risk (VHR) to high risk (HR) with increasing pyrolysis temperatures. The results indicate that the risk index ($E_{r}^{i}$) values of Zn, Cu, Ni, Mn, Cr and As in WSM and WSMB were all less than 5, suggesting low risk (LR) to the environment. Similarly, the risk indices of Cd in WSM and WSMB changed from high risk (HR) to considerable risk (CR) with rising pyrolysis temperatures. Therefore, Cd in CM, WSM and their biochars should be of major concern. The PERI values of CM and WSM were 42.59 and 44.73, respectively. The results indicated that the heavy metals in CM and WSM were at a moderate risk level. The PERI values of CM, WSM and their biochars decreased from 42.59 to 26.91 and from 44.73 to 16.78 after pyrolysis, thus reducing the underlying ecological risk of heavy metal in biochars from moderate risk (MR) to low risk (LR). However, the PERI values for both CMB and WSMB showed moderate risk at pyrolysis temperatures ranging from 200 to 500 °C and 200 to 650 °C, respectively. When the pyrolysis temperature reached 800 °C, the PERI value of CMB and WSMB showed low risk. Consequently, pyrolysis at 800 °C can provide an effective way to lessen the direct and underlying heavy metal toxicity of biochars produced from manures. In conclusion, pyrolysis can be used as an effective method to mitigate the environmental toxicity and potential ecological risks of HMs.

### Risk assessment code of heavy metals in manures and their biochars

Chemical speciation of HMs is directly correlated with their bioavailability and eco-toxicity in the environment^28,36^. The risk assessment code (RAC) was implemented to identify the environmental risks of HMs in CM, WSM, CMB and WSMB. The risk assessment results for CM, WSM and their biochars are shown in Table 6. Overall, except for Cu in CMB, the RAC values of Zn, Ni, Mn, Cr, As and Cd in CM, WSM and their biochars decreased when pyrolysis temperatures rose from 200 to 800 °C. The highest RAC value of Cu in biochars was found at 200 °C and decreased with increasing pyrolysis temperatures. Previous research found that Cu was mostly bound with organic groups (e.g., Cu-cysteine and Cu-citrate) in the biochars produced at the low pyrolysis temperature, whereas the percentage of residual Cu (CuO) significantly increased with the increasing pyrolysis temperature^30^. Therefore, Cu in CMB at 200 °C should attract widespread attention.

**Table 6:** Risk assessment code (RAC) of heavy metals in CM, WSM, CMB and WSMB.

Sample	HM (%)						
	Zn	Cu	Ni	Mn	Cr	As	Cd
CM	12.66 ± 0.12(MR)	3.24 ± 0.14(LR)	8.43 ± 0.01(LR)	15.34 ± 0.02(MR)	12.48 ± 0.01(MR)	22.84 ± 0.02(MR)	0.68 ± 0.01(NR)
CM200	5.91 ± 0.01(LR)	5.06 ± 0.01(LR)	2.77 ± 0.02(LR)	12.17 ± 0.01(MR)	5.29 ± 0.02(LR)	12.59 ± 0.12(MR)	0.72 ± 0.03(NR)
CM350	2.03 ± 0.02(LR)	4.20 ± 0.01(LR)	3.81 ± 0.11(LR)	8.12 ± 0.02(LR)	0.96 ± 0.01(NR)	17.69 ± 0.21(MR)	0.19 ± 0.01(NR)
CM500	0.65 ± 0.02(NR)	4.48 ± 0.02(LR)	4.50 ± 0.21(LR)	8.23 ± 0.20(LR)	0.59 ± 0.03(NR)	8.53 ± 0.11(LR)	0.41 ± 0.02(NR)
CM650	1.30 ± 0.01(LR)	4.00 ± 0.01(LR)	2.53 ± 0.14(LR)	7.58 ± 0.10(LR)	0.44 ± 0.02(NR)	10.37 ± 0.02(MR)	0.45 ± 0.00(NR)
CM800	0.35 ± 0.02(NR)	3.91 ± 0.01(LR)	3.75 ± 0.21(LR)	5.94 ± 0.13(LR)	1.21 ± 0.01(LR)	6.87 ± 0.01(LR)	0.53 ± 0.01(NR)
WSM	26.06 ± 1.20(MR)	8.03 ± 0.01(LR)	13.52 ± 0.11(MR)	19.99 ± 1.20(MR)	1.54 ± 0.02(LR)	13.50 ± 0.01(MR)	17.94 ± 0.01(MR)
WSM200	25.91 ± 1.10(MR)	5.72 ± 0.02(LR)	5.91 ± 0.10(LR)	18.03 ± 1.12(MR)	1.62 ± 0.01(LR)	14.17 ± 0.02(MR)	15.61 ± 0.01(MR)
WSM350	26.12 ± 0.01(MR)	2.10 ± 0.10(LR)	2.33 ± 0.02(LR)	16.35 ± 1.20(MR)	1.61 ± 0.02(LR)	5.48 ± 0.02(LR)	17.98 ± 0.23(MR)
WSM500	24.01 ± 0.02(MR)	0.48 ± 0.02(NR)	1.87 ± 0.12(LR)	12.82 ± 0.01(MR)	0.54 ± 0.00(NR)	3.36 ± 0.02(LR)	5.58 ± 0.12(LR)
WSM650	17.98 ± 0.03(MR)	0.84 ± 0.12(NR)	1.78 ± 0.12(LR)	13.09 ± 0.02(MR)	0.99 ± 0.03(NR)	2.94 ± 0.12(LR)	6.28 ± 0.14(LR)
WSM800	9.92 ± 0.01(LR)	0.21 ± 0.01(NR)	3.77 ± 0.02(LR)	13.20 ± 0.01(MR)	0.84 ± 0.01(NR)	2.91 ± 0.01(LR)	2.21 ± 0.27(LR)

Note: HM: heavy metal, Zn: zinc, Cu: copper, Ni: nickel, Mn: manganese, Cr: chromium, As: arsenic, Cd: cadmium, CM: chicken manure, WSM: water-washed swine manure, CMB: chicken manure biochar, WSMB: water–washed swine manure biochar, CM200-CM800: chicken manure biochar converted by pyrolysis temperatures from 200 to 800 °C, WSM200-WSM800: water-washed swine manure biochar converted by pyrolysis temperatures from 200 to 800 °C.

The heavy metals in the sample can be categorized by RAC as no risk (NR), low risk (LR), medium risk (MR) high risk (HR), and very high risk (VHR), with RAC value scopes of < 1%, 1–10%, 10 – 30%, 30 – 50%, and > 50%, respectively.

The RAC values of As, Mn, Zn and Cr in CM were 22.84, 15.34, 12.65 and 12.47%, respectively, which indicated that As, Mn, Zn and Cr in CM had MR when CM was applied to the environment. After pyrolysis, the RAC values of As, Mn, Zn and Cr in CMB were reduced to 6.86, 5.94, 0.34 and 1.21%, respectively, which indicated that As, Mn, Zn and Cr in CMB had LR, LR, NR and LR, respectively, when CMB was applied to the soil. The RAC values of Ni and Cu in CMB at 800 °C were 3.75 and 3.90%, respectively, which indicated LR when CMB was applied to the environment. The RAC values of Cd in CM and CMB indicated NR. The RAC values of Zn, Mn, Cd, As and Ni in WSM were 26.06, 19.99, 17.93, 13.49 and 13.51%, respectively, which indicated MR to the environment. With pyrolytic temperature increases from 200 to 800 °C, the RAC values of Zn, Mn, Cd, As and Ni in WSMB were reduced to 9.92, 13.19, 2.20, 2.90 and 3.77%, respectively which indicated MR, MR, LR, LR and LR, respectively. The RAC values of Cu and Cr in WSMB indicated no risk to the local environment. The results suggest that pyrolysis is beneficial to lessen the environmental risk of HMs in CMB and WSMB at 800 °C.

The results show that the environmental risk of HMs in biochars decreased with pyrolytic temperature increases from 200 to 800 °C. After pyrolysis as biochars, it is environmentally friendly because pyrolysis can address HMs and transform unstable fractions into stable fractions^39,54^.

## Conclusion

With pyrolysis temperature increases from 200 to 800 °C, the heavy metal concentrations in CMB and WSMB were all greater than those in CM and WSM. The remaining ratios of HMs decline owing to the decomposition of organic substances and ash trapped or reacted with HMs. The chemical speciation of HMs in biochars demonstrated that the percentage of the bioavailable portion (e.g., F1 + F2) declined and the percentage of the residual portion (F4) stable increased with increasing pyrolysis temperature. It demonstrated that HMs to form stable fractions in the residue with increasing purolysis temperature. In both CMB and WSMB, the proportion of F3 increases with increasing pyrolysis temperature. It demonstrated that the complexation of HMs and the fixation of HMs with organic matter. As the pyrolysis temperature continues to rise, the proportion of F3 decreased and the F4 increased. It can ascribe to HMs, at high temperature, having adequate energy for breaking the associated bonds to convert the F3 to the F4. Therefore, pyrolysis effectively converts the unstable portion of HMs in biochar into a stable portion, reducing the HMs availability to plants and leaching risk.

According to the result of GAI, PERI and RAC, the contamination level was reduced with increasing pyrolysis temperature. However, the contamination level of Cd in CM, WSM and their biochars still suggested that very high risk and considerable risk to the local environment. Therefore, Cd in CM, WSM and their biochars should be of major concern in the next application.

In conclusion, pyrolysis can provide an effective way to lessen the direct and underlying heavy metal toxicity of biochars produced from manures. Furthermore, pyrolysis at 800 °C can provide an effective way to lessen the direct and underlying heavy metal toxicity of biochars produced from manures. In the future, a proper catalyst can be research during the pyrolysis of CM and WSM to further decrease concentration and bioavailability of heavy metals.

## Methods and materials

### Specimen collection and pretreatment

This research chose chicken manure (CM) and water-washed swine manure (WSM) as specimens of raw materials. Chicken manure was obtained from a chicken farm outside Ningxiang County, Changsha city, Hunan Province. At the same time, water-washed swine manure was collected in the solid matter separated by a solid–liquid separator from a large-scale piggery outside of Nanchang, Jiangxi Province. The collected samples were dried at a fixed temperature (60 °C). The dried samples were crushed and sieved (100 mesh).

### Pyrolysis process

**Figure 5:**
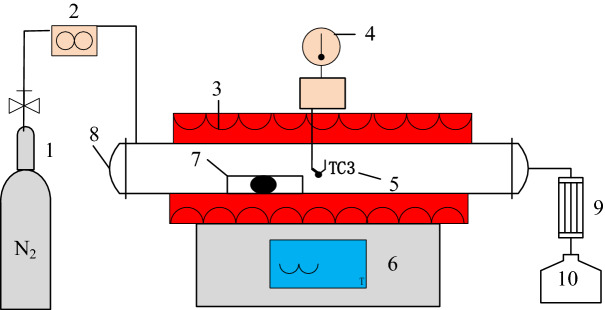
Schematic diagram of laboratory-scale pyrolyser. Note: 1. Cylinder of N2; 2. Rotameter; 3. Heating chamber; 4. Temperature measure device; 5. Thermocouple; 6. Electronic temperature controller; 7. Quartz boat; 8. Quartz tube; 9. Condenser; 10. Collector.

The samples were pyrolysis in dedicated laboratory-scale pyrolyzers (SK-G08123K, China), as shown in Fig. 5. CM and WSM were utilized for the laboratory pyrolysis experiment. The laboratory-scale pyrolyzer was composed of a quartz boat (inner diameter = 72 mm, length = 1000 mm), a heating chamber with an electronic temperature controller, a quartz tube, a condenser and a collector. The temperature distribution was obtained by a thermocouple placed in the center of the reactor. During each pyrolysis, 5 g of dried manure will be put into the quartz boat. Pure nitrogen was then pumped into the quartz boat and the heating furnace for 10 min. The entire pyrolysis process maintains a 200 ml/min pure nitrogen purge. The pyrolysis temperatures were set to 200, 350, 500, 650 and 800 °C. The rate of pyrolysis temperature increase was 10 °C/min, and each specified temperature lasted for one hour.

The CM and WSM biochars were crushed with a mortar and sifted into parts with particle sizes of 100, which were referred to as chicken-manure-derived biochar (CMB) and water-washed swine manure-derived biochar (WSMB).

### Analytical processes

#### Physical and chemical analysis

Elemental analysis of CM, WSM and manure-derived biochar was performed with a CHNOS Elemental Analyzer Vario EL III (Elementary Analysis systems GmbH, Germany). Proximate analysis was performed according to correlative criteria (GB/T28731-2012). The recovery ratio for HMs in the materials (e.g., CM, WSM and their biochars) was approximately 90–105%. Additionally, blank and duplicate samples were analyzed, and the analysis results were standardized. The relative standard deviations (RSD) of all repeat samples measured in this experiment were less than 10%.

#### Sequential extraction process of heavy metals

BCR extraction was utilized to analyse the chemical distributions of HMs in CM, WSM and manure-derived biochar^55^. A modified BCR extraction is described as follows: acid extractable (F1), reducible (F2), oxidizable (F3), and residual (F4).

#### The heavy metals available to plants

According to previous studies, diethylenetriamine pentaacetic acid (DTPA) can be used to determine those HMs that are available to plants^56^. The extractable portion of DTPA was mechanically oscillated by 25 g CMB and WSMB samples at 25 ℃ for 2 h, 50 ml 0.005 mol l^−l^ DTPA, 0.01 mol l^−l^ CaCl_2_, 0.1 mol l^−1^ triethanolamine buffered pH 7.3. At the same time, 0.1 mol l^−1^ HCl was leached at 25 ℃ to obtain 0.1 mol l^−1^ HCl leachable part. Leaching at a solid–liquid ratio of 1:5 for 1.5 h. Quantitative filter paper is used to filter suspension.

#### Leaching rates of heavy metals

According to previous studies, the results of the toxicity characteristic leaching procedure (TCLP) can be utilized to identify the leaching rates of HMs in CM, WSM, CMB and WSMB^3^. The sample (1 g) was mixed with 20 ml glacial acetic acid extract (1:20 ratio) at a pH of 2.88 ± 0.05 and stirred at 120 rpm for 20 h. The compound was then synthesized and centrifuged for 20 min at 4000 revolutions per minute, with the upper fluid passing through a 0.45 m membrane filter. Each experiment is in triplicate.

#### Remaining ratio

The remaining ratio (R) is determined as the amount of HMs in CMB and WSMB and HMs in CM and WSM; R was computed using the following equation:

1 $$ {\text{R}}\left( \% \right) = {\text{B}}_{{\text{X}}} \times {\text{Y}}/{\text{TM}}_{{\text{X}}} \times {1}00 $$

where X: one variety of HMs; TB_X_: total HMs content in CNB and WSMB (mg kg^−1^); Y: output of biochars; and TM_X_: total HMs content in the sample (mg kg^−1^).

#### Environmental risk assessment

By calculating PERI, the potential ecological risks index from HMs in biochar samples were assessed. The PERI calculation formula is as follows:

2 $$ C_{f}^{i} = C_{D}^{i} /C_{R}^{i} $$

3 $$ E_{r}^{i} = T_{r}^{i} \times C_{f}^{i} $$

4 $$ PERI = \sum\limits_{i = 1}^{n} {E_{r}^{i} } $$

where $C_{f}^{j}$ is the contamination coefficient of HMs, $C_{D}^{i}$ is the concentration of HMs in CM, WSM, CMB and WSMB, $C_{r}^{i}$ is the background concentration of HMs and is defined as Bn, $T_{r}^{i}$ is the potential ecological index of HMs, and PERI is the potential ecological risk induced by the total contamination. Based on Hakanson, the potential ecological indices ($T_{r}^{i}$) for Zn, Cu, Ni, Mn, Cr, As and Cd used in this study were 1, 5, 5, 1, 2, 10 and 30 mg/kg, respectively. The soil background contents of Zn, Cu, Ni, Mn, Cr, As and Cd used in this research were 90, 25, 30, 380, 64, 14 and 0.08 mg/kg, respectively. To accurately express pollution levels and potential ecological risks from HMs in CM, WSM, CMB and WSMB, the total acid extractable (F1) and reducible (F2) amounts for individual HMs were chosen instead of the total concentration to evaluate the GAI and PERI. $E_{r}^{i}$ < 5 indicates Low Risk (LR), 5 < ${\mathrm{E}}_{\mathrm{r}}^{\mathrm{i}}$ $E_{r}^{i}$ < 10 indicates Moderate Risk (MR), 10 < $E_{r}^{i}$ < 20 indicates Considerable Risk (CR), 20 < $E_{r}^{i}$ < 40 indicates High Risk (HR), and $E_{r}^{i}$ > 40 indicates Very High Risk (VHR). In this research, PERIs below 30 indicate Low Risk (LR), a PERI range from 30 to 60 indicates Moderate Risk (MR), a PERI range from 60 to 120 indicates Considerable Risk (CR), and PERIs greater than 120 indicates Very High Risk (VHR)^43^.

#### Risk assessment code

The risk assessment code is commonly chosen to evaluate HM pollution in precipitates and soils^57,58^. In this study, RAC assesses the availability of HMs in CM, WSM, CMB and WSMB by using a scale of the percentage of HMs present in the acid extractable (F1) fraction. A ratio of HMs in F1 less than 1% indicates No Risk (NR); a ratio in the range of 1% to 10% indicates Low Risk (LR); a ratio in the range of 10% to 30% indicates Medium Risk (MR); a ratio in the range of 30% to 50% indicates High Risk (HR); and a ratio greater than 50% indicates Very High Risk (VHR)^59^.
